# Real-World Safety of Concurrent Measles–Mumps–Rubella and Varicella Vaccination in Korean Infants: A Multicenter Self-Controlled Case Series Study

**DOI:** 10.3390/vaccines14070553

**Published:** 2026-06-24

**Authors:** Sujin Choi, Bin Ahn, Yeonjoo Lee, Gwanglok Kim, Young June Choe, Youn Young Choi

**Affiliations:** 1 Department of Pediatrics, Konkuk University Medical Center, Seoul 05030, Republic of Korea; 20250295@kuh.ac.kr; 2 Department of Pediatrics, Seoul Metropolitan Government–Seoul National University Boramae Medical Center, Seoul 07061, Republic of Korea; bin325@snu.ac.kr; 3 Department of Pediatrics, Seoul National University College of Medicine, Seoul 03080, Republic of Korea; 4 Regulatory Science & Product Development Division, GC Biopharma Corporation, Yongin 16924, Republic of Korea; nayj@gccorp.com (Y.L.); kimgr@gccorp.com (G.K.); 5 Department of Pediatrics, Korea University College of Medicine, Seoul 02841, Republic of Korea; choey@korea.ac.kr

**Keywords:** varicella vaccine, measles-mumps-rubella vaccine, vaccine safety

## Abstract

Background: Measles, mumps, rubella (MMR) and varicella vaccines are often co-administered to optimize coverage, yet safety concerns regarding febrile convulsions persist. In South Korea, MMR and varicella vaccines are administered as separate injections during a single visit (MMR + V). This study evaluated the real-world safety of concurrent MMR + V vaccination, focusing on the domestically implemented MAV/06 and Oka-derived strains. Methods: We conducted a multicenter self-controlled case series (SCCS) study of children aged 12–23 months who received MMR + V and hepatitis A vaccine (HAV) between 2015 and 2024. Using electronic health records, we identified predefined adverse events (AEs), including fever and healthcare visits. Adjusted relative risks (aRRs) were estimated using conditional Poisson regression. Results: Among 3035 children (52.3% male; median age, 12 months), 71.7% received the MAV/06 varicella strain. A distinct peak in AEs occurred 7–13 days after MMR + V administration, with fever showing the greatest increase (aRR, 4.27; 95% CI, 2.76–6.60). The risks of total sick visits (aRR, 2.15; 95% CI, 1.70–2.71) and acute care visits (aRR, 2.13; 95% CI, 1.46–3.10) were similarly confined to this interval and returned to baseline thereafter. Febrile convulsions were uncommon (aRR, 5.37; 95% CI, 1.20–24.01). No excess risks were observed during the HAV or overlap periods, and no synergistic effects of intensive multi-vaccine administration were detected. Conclusions: Concurrent administration of MMR and varicella vaccines in Korean infants—predominantly using the MAV/06 strain—was associated only with expected, transient increases in fever during days 7–13 postvaccination. No serious or sustained safety signals were identified, supporting the continued use of Korea’s separate-injection MMR + V strategy.

## 1. Introduction

Measles, mumps, rubella, and varicella are highly contagious childhood diseases that can cause substantial morbidity and mortality in the absence of effective immunization. To optimize vaccine uptake and reduce healthcare visits, global immunization guidelines—including those from the U.S. Centers for Disease Control and Prevention [1]—recommend either the quadrivalent measles–mumps–rubella–varicella (MMRV) vaccine or concurrent administration of the trivalent measles–mumps–rubella (MMR) and monovalent varicella vaccines (MMR + V).

Although combination vaccines offer greater convenience, safety concerns regarding febrile convulsions persist. In the United States, children aged 12–23 months who received their first dose as MMRV had nearly a twofold higher risk of febrile convulsions within 7–10 days than those who received separate MMR and varicella injections [2,3]. MMRV was estimated to cause one additional febrile convulsion per 2300–2600 doses administered [2,3], whereas the separate MMR + V regimen was associated with a lower relative risk. This finding suggests that administering antigens via separate injections may attenuate the acute inflammatory response [4,5].

The Republic of Korea (hereafter Korea) provides a distinctive setting for evaluating the MMR + V strategy. Korea has never adopted the combined MMRV vaccine; instead, MMR and varicella have been routinely co-administered as separate injections at the 12-month visit, maintaining vaccination coverage at 97.3%. This rate is among the highest in the world and exceeds reported primary series completion in many other developed nations by 2–10% [6]. In addition, the Korean national immunization program (NIP) incorporates both Oka-derived and domestically developed MAV/06 varicella strains; however, real-world safety data within the concentrated 12-month immunization window remain limited.

Using a self-controlled case series (SCCS) design, we evaluated the real-world safety of MMR + V vaccination under routine clinical conditions in Korea. Subgroup analyses focused on the MAV/06 strain [7,8]—previously unique to the Korean NIP but now adopted in select other countries—to provide evidence applicable beyond Korea [7,8]. Risk patterns were further examined within the 12-month immunization window, accounting for potential temporal overlap with subsequent inactivated vaccines, such as the hepatitis A vaccine (HAV).

## 2. Materials and Methods

### 2.1. Participants and Setting

We conducted a multicenter SCCS study at three teaching hospitals in Seoul, Korea (Boramae Medical Center, Konkuk University Medical Center, and Korea University Anam Hospital), between 2015 and 2024. The cohort included infants aged 12–23 months who received concurrent administration of the first dose of MMR (M-M-R^®^ II; Merck & Co., Inc., Rahway, NJ, USA) and varicella vaccines (MMR + V) and who also received the first dose of HAV at the same institution. HAV was selected as an active comparator because its first dose is routinely administered within the same 12-month immunization window. Recipients of either Havrix (GlaxoSmithKline, London, UK) or Vaqta (Merck & Co., Inc., Rahway, NJ, USA) were analyzed as a single HAV group because both are inactivated vaccines with comparable safety profiles; together, they represent background healthcare utilization within the same 12-month immunization window.

Electronic health record (EHR) data were used to identify baseline comorbidities using the Pediatric Complex Chronic Condition (CCC) system version 3 [9]. The CCC framework encompasses conditions affecting neurological, cardiovascular, respiratory, renal, gastrointestinal, hematologic, metabolic, malignancy, and neonatal systems. Children were classified as having a CCC if the corresponding International Classification of Diseases, Tenth Revision (ICD-10) codes appeared on two or more separate occasions in their EHR during the study period. Predefined AEs—fever, sick visits, febrile convulsions, allergic reactions, and rash—were identified using diagnostic criteria described below and detailed in the Supplementary Methods. Sick visits were categorized by healthcare setting to distinguish routine from urgent utilization. Total sick visits included all medical encounters—outpatient visits, emergency department (ED) visits, and hospitalizations—coded with predefined acute illness ICD-10 codes (Supplementary Table S1). To isolate higher-acuity events, acute care visits were restricted to ED encounters and hospitalizations. To ensure event independence and avoid overestimation due to follow-up care, only the first encounter of an independent episode (defined by a 42-day washout period) was counted as an outcome.

For comprehensive surveillance, EHR data were supplemented with an independent review of spontaneous reports from the Korea Adverse Event Reporting System (KAERS) at each participating center.

### 2.2. Outcome Definitions

Febrile convulsions were identified using ICD-10 code R56.0, or codes R56.8/R56.8 A when recorded in conjunction with a concurrent fever code. Given known variability in case definitions across surveillance systems, a broad code-based definition was applied to maximize sensitivity. Allergic reactions were defined as encounters coded with urticaria (L50), anaphylaxis (T78.0–T78.2), or other allergic responses (T78.4). Fever, rash, and sick visits were defined using ICD-10 codes detailed in Supplementary Table S1.

### 2.3. Study Design and Risk Periods

The observation window extended from 90 days before the index date—defined as the date of the first vaccination event (either MMR + V or HAV)—to 180 days after the subsequent vaccination.

To identify the peak risk period associated with live-attenuated viral replication, we first performed a temporal trend analysis. The post-MMR + V period was subdivided into weekly intervals (0–6, 7–13, 14–20, 21–27, and 28–42 days), with the 7–13-day window specifically defined as the expected period of vaccine-induced immune response [10,11].

**Figure 1:**
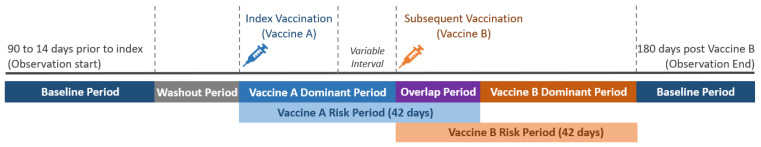
Study scheme and exposure states for the self-controlled case series analysis. The schematic illustrates the SCCS study design for a representative participant. In this study, Vaccine A represents the first administered dose between MMR + V and HAV, whereas Vaccine B represents the subsequent dose of the alternate vaccine. The index date is defined as the administration date of Vaccine A. The total observation period extends from 90 days before the index date to 180 days after administration of Vaccine B. Person-time within this window was categorized into four mutually exclusive exposure states: MMR + V-dominant, HAV-dominant, Overlap, and Baseline periods. Although MMR and varicella vaccines are administered as separate injections, they are represented as a single vaccine exposure (either Vaccine A or Vaccine B) in this scheme.

To further distinguish vaccine-specific effects and account for the intensive 12-month immunization schedule, person-time was classified into four mutually exclusive states (Figure 1):

1. MMR + V-dominant period: Days 0–42 following concurrent administration of MMR and varicella vaccines, excluding any HAV exposure.
2. Overlap period: Days during which the 42-day risk windows following MMR + V and HAV overlapped.
3. HAV-dominant period: Days 0–42 following HAV administration, excluding any MMR + V exposure.
4. Baseline period: All remaining observation time, excluding a 14-day prevaccination washout period, which served as the within-person reference period.

### 2.4. Statistical Analysis

Vaccine safety was evaluated using the SCCS method, which inherently controls for fixed individual-level confounders—including sex and the presence of a CCC—by using each participant as their own control. Adjusted relative risks (aRRs) were estimated using conditional Poisson regression models fitted with the gnm package, with an offset for the logarithm of person-days [12], restricted to informative cases (i.e., individuals with ≥1 event during follow-up).

The analysis was conducted in two stages with distinct objectives. First, a temporal trend model compared weekly post-MMR + V intervals against the Baseline period to characterize the temporal pattern of AE risk and establish biological plausibility for vaccine-attributable reactogenicity; this model was adjusted for age (in months), season, and the number of concomitant vaccines. Second, an exposure state model assessed MMR + V safety against both a vaccine-free reference (Baseline) and two active comparators—the Overlap and HAV-dominant period—to determine whether MMR + V confers excess risk beyond routine 12-month inactivated vaccine administration and whether synergistic effects exist during the Overlap period. In addition to the covariates adjusted in the first model, this model incorporated the pandemic period (2015–2019, 2020–2022, and 2023–2024) to account for long-term temporal shifts in healthcare utilization. Direct comparisons between exposure states (e.g., MMR + V-dominant vs. HAV-dominant) were derived as ratios of aRRs from the same model, with 95% CIs calculated using the delta method.

All analyses were conducted using R version 4.4.3 (R Foundation for Statistical Computing, Vienna, Austria). Continuous variables are presented as medians with interquartile ranges (IQRs), whereas categorical variables are expressed as frequencies and percentages [12]. Statistical significance of individual coefficients was assessed using two-sided Wald tests, with *p* < 0.05 as the threshold.

The study was approved by the Institutional Review Boards of all participating centers (Lead Institution IRB No. 2025 AN0399).

## 3. Results

### 3.1. Patient Characteristics

The study cohort consisted of 3035 children (52.3% male) with a median age of 12 months (IQR, 12–12) at the time of MMR + V vaccination (Table 1). CCCs were present in 13.6% (n = 412) of the cohort, with neonatal conditions, including extreme prematurity, accounting for 53.2% of these cases. During the observation period, participants received a median of 7 vaccine doses (IQR, 7–8) and had a median of 9 healthcare encounters (IQR, 6–14). These encounters were primarily outpatient visits, reflecting high continuity of care within the participating institutions.

### 3.2. Immunization Patterns and Intensity

MMR + V was the primary entry point into the 12-month immunization series in 79.6% of children. Most received MMR + V followed by HAV (91.9%), with a median inter-vaccine interval of 17 days (IQR, 7–63); 44.7% received both vaccines within 14 days. Additional vaccines were co-administered at the MMR + V visit in 39.1% of children, most commonly the Japanese encephalitis vaccine (37.0%). MAV/06 was the predominant varicella strain (71.7%), and vaccinations were evenly distributed across seasons.

High-density vaccine exposure was common, with children receiving a median of 6 vaccinations (IQR, 5–7) during the 42-day post-MMR + V risk period. Vaccine intensity remained comparable across exposure states, with a median of 3 vaccinations during both the MMR + V-dominant (IQR, 2–5) and Overlap (IQR, 2–4) periods. The majority of vaccinations (61.0%) were administered during the prepandemic period (2015–2019).

### 3.3. Temporal Trends and Peak Risk Periods

aRRs were evaluated in weekly intervals following MMR + V administration, focusing on outcomes with sufficient event counts: fever, total sick visits, and acute care visits (defined as ED encounters and hospitalizations).

**Figure 2:**
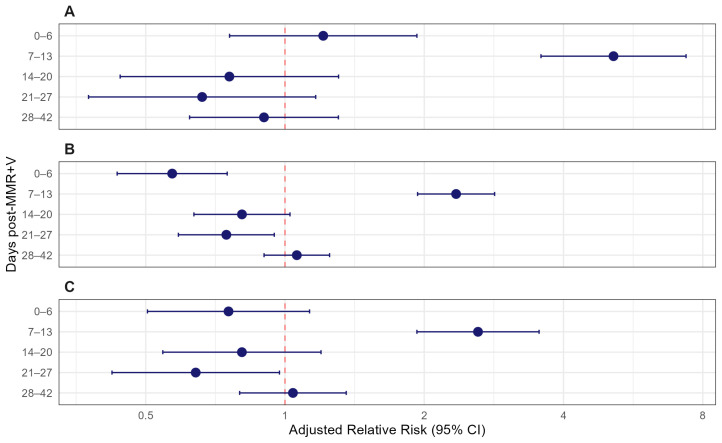
Temporal trends in adjusted relative risks for major clinical outcomes following MMR + V vaccination. The forest plots illustrate the aRRs and 95% confidence intervals (CIs) for (**A**) fever, (**B**) total sick visits, and (**C**) acute care visits (emergency department visits or hospitalization) during subdivided intervals of the MMR + V risk period. All risks were compared with the Baseline period and adjusted for age, season, and the number of concomitant vaccines. The dashed vertical line represents the reference value (aRR = 1.0). Abbreviations: aRR, adjusted relative risk; CI, confidence interval; MMR + V, concurrent measles–mumps–rubella and varicella vaccination.

A distinct and statistically significant peak in risk was observed during days 7–13 following MMR + V vaccination (Figure 2 and Supplementary Table S2). Fever demonstrated the most pronounced clustering during this interval (aRR, 4.27; 95% CI, 2.76–6.60; *p* < 0.001), whereas risks during the immediate (0–6 days) and later (14–42 days) periods remained at baseline levels. Total sick visits (aRR, 2.15; 95% CI, 1.70–2.71; *p* < 0.001) and acute care visits (aRR, 2.13; 95% CI, 1.46–3.10; *p* < 0.001) followed a similar pattern, with significant elevations confined to the 7–13-day window and a return to baseline after day 14.

Febrile convulsions also exhibited a transient increase during the 7–13-day window (aRR, 5.37; 95% CI, 1.20–24.01; *p* = 0.028), although the wide confidence intervals reflect the rarity of these events (Supplementary Table S2). Rash risk likewise peaked during the same interval (aRR, 5.29; 95% CI, 1.56–17.94) and remained significantly elevated through day 20 (aRR, 5.23; 95% CI, 1.60–17.08).

Subgroup analyses restricted to the MAV/06 strain recipients and children with any CCC demonstrated consistent temporal patterns. Among this chronic disease cohort, the risk of fever remained concentrated within the 7–13-day window, mirroring the primary findings. Notably, although febrile risks were elevated, no corresponding increase in total sick or acute care visits was observed in this subgroup during this peak period.

### 3.4. Comparative Risks Across Exposure States

We further compared the MMR + V-dominant period with other exposure states to determine whether risks were exacerbated by the intensive 12-month immunization schedule (Table 2).

Overall, the comparative analysis indicated that the safety profile of MMR + V remained within clinically predictable limits. For fever, although the MMR + V-dominant period showed a significant elevation relative to baseline (aRR, 2.43; 95% CI, 1.57–3.74; *p* < 0.001), no corresponding increase was observed during the HAV-dominant or Overlap periods. Notably, for total sick visits and acute care visits, the risk during the MMR + V-dominant period did not differ significantly from baseline (aRR, 1.16; *p* = 0.192 and aRR, 1.09; *p* = 0.649, respectively).

When directly comparing exposure windows, total sick visits were more frequent during the MMR + V-dominant period than during the HAV-dominant period (aRR, 1.43; *p* = 0.009). Febrile convulsions, allergic reactions, and rash were rare, and although event counts were numerically higher during the MMR + V-dominant period, aRRs did not reach statistical significance across any vaccination state. Importantly, there was no evidence of synergistic risk during the Overlap period for any outcome.

These findings remained consistent in subgroup analyses. For the MAV/06 strain, no significant deviations from the primary findings or synergistic risks during the Overlap period were observed—with febrile convulsion risks comparable to or lower than those in the overall cohort (Supplementary Table S3). Similarly, among children with any CCC, the risk of fever was elevated during the MMR + V-dominant period (aRR, 3.06; *p* = 0.079) but did not result in increased healthcare utilization, as both total and acute care visits remained stable across all exposure states.

Finally, an independent secondary review of institutional KAERS reports identified no spontaneous AEs related to MMR or varicella vaccines among cohort participants.

## 4. Discussion

This multicenter SCCS study of more than 3000 Korean children demonstrates that AEs following MMR + V co-administration were generally mild and transient. Fever and healthcare utilization were strictly confined to the 7–13-day postvaccination window, with risks returning to baseline thereafter. No significant increases in serious outcomes, such as febrile convulsions or acute care visits, were observed.

Our findings indicate that although the risk of fever increased compared with both the baseline and the active comparator, this elevation was confined to the 7–13-day window. The magnitude of risk (aRR, 4.27 during days 7–13; aRR, 2.43 for the MMR + V-dominant period) aligns with previously reported risks for MMR-only vaccination, including an aRR of 4.3 (7–10 days) reported by the U.S. Vaccine Safety Datalink [2] and an aRR of 3.28 (95% CI, 2.23–4.82) within three weeks of MMR administration [11]. These findings suggest that the observed febrile reactogenicity is largely attributable to the measles component, which is considered the most pyrogenic element of the combination vaccine [13]. The 7–13-day peak risk aligns with attenuated measles virus replication [2,4,5,10,11], whereas varicella-related viremia typically occurs later and rarely exhibits such sharp temporal clustering [13,14]. Indeed, prior studies have confirmed that varicella vaccination alone does not produce significant temporal clustering of fever [2,14,15,16].

Consistent with the literature demonstrating minimal risk differences between MMR alone and MMR + V [2,17], our data suggest that the clustering of fever and healthcare utilization reflects an expected physiological response to MMR rather than a synergistic effect of co-administration. This interpretation is further supported by a phase III RCT demonstrating comparable reactogenicity profiles for MMR co-administered with or without varicella vaccine, with fever as the predominant solicited adverse event, though that trial evaluated second-dose administration in older children [18]. Importantly, the absence of heightened risk compared with the Overlap period indicates that MMR + V does not pose a disproportionate safety burden within the intensive 12-month immunization schedule.

Given that post-vaccination febrile convulsions are typically precipitated by vaccine-induced fever [19], measles-containing vaccines (MCVs) are well-established triggers of febrile convulsions during a window of attenuated viral replication—a risk predominantly attributable to the measles component [2,4,5]. The absolute attributable risk of febrile convulsions following MCV, while statistically significant, remains low relative to the background rate of febrile convulsions in young children, which occur in approximately 2–5% of children aged 6 months to 5 years [20]. Within this context, the separate-injection MMR + V strategy provides a highly favorable safety profile by delivering the necessary immunizations without compounding the expected baseline reactogenicity of the individual components [2].

In our cohort, febrile convulsions were uncommon, and the small event count precludes firm conclusions. Nonetheless, the absence of a significantly elevated febrile convulsion risk during the MMR + V-dominant period relative to both the HAV-dominant and Overlap periods provides preliminary supporting evidence that concurrent MMR + V administration does not markedly increase febrile convulsion risk beyond that attributable to routine 12-month vaccinations. This finding is consistent with the established safety evidence that neither varicella vaccination alone nor MMR + V co-administration is associated with increased febrile seizure risk beyond that attributable to the measles component—including after adjustment for concomitant MMR administration [2,21,22]. Of particular note, no strain-specific signal was observed in our predominantly MAV/06 subgroup, suggesting that this favorable profile extends to the MAV/06 strain.

Healthcare utilization patterns further underscore the stability of the intensive 12-month immunization schedule. The reduction in sick visits during the HAV-dominant period likely reflects a healthy vaccinee effect, whereby vaccinations are preferentially scheduled during periods of clinical stability. Among children with any CCC, healthcare utilization did not increase despite an elevated febrile peak, suggesting that expected reactogenicity does not destabilize high-risk groups, likely due to high baseline medical supervision and proactive parental education.

Our findings are particularly relevant to Korea’s dual-strain varicella vaccination program. Since 2005, the domestically developed MAV/06 strain has been implemented within the NIP alongside various Oka-derived vaccines. With more than 70% of the cohort receiving the MAV/06 strain, the absence of clinically significant safety signals suggests that its general safety and tolerability profile is comparable to established global benchmarks [23,24]. Although real-world safety data for MAV/06 during co-administration have been limited, our findings address this gap and support the long-term sustainability of this unique dual-strain, separate-injection (MMR + V) policy, which consistently maintains nearly 97% vaccination coverage and serves as a viable model for achieving herd immunity [6,25].

The robustness of these findings is supported by several methodological strengths. The SCCS design inherently controls for all fixed individual-level confounders by using each child as their own control, minimizing selection bias and confounding. Incorporating HAV as an active comparator accounts for age-specific healthcare-seeking behaviors, ensuring that observed signals are vaccine-specific. The detailed weekly risk interval analysis further establishes biological plausibility, with the distinct clustering of events within the 7–13-day window confirming a direct physiological response rather than a chance occurrence.

Nevertheless, several limitations should be acknowledged. First, reliance on EHR data from the participating centers may have underestimated encounters at outside institutions; however, the high continuity of care—reflected by a median of 7 vaccine doses and 9 healthcare encounters per participant—suggests that most clinically significant encounters were captured. Furthermore, AE ascertainment depended entirely on ICD-10 codes assigned by treating clinicians, which are subject to coding variability and potential undercoding, and lack structured diagnostic criteria or objective thresholds. Second, minor AEs managed at home may have been underreported. Finally, the rarity of allergic reactions, rash, and febrile convulsions limited the statistical power for these outcomes, though the absence of significant safety signals for serious events supports a safety profile consistent with international benchmarks.

## 5. Conclusions

In conclusion, concurrent administration of MMR and varicella vaccines—the latter of which predominantly utilized the MAV/06 strain—aligns with international safety data and is associated only with expected, short-lived increases in fever and healthcare utilization. These findings provide robust real-world evidence supporting the continued use of this separate-injection framework in high-coverage settings and affirm the safety of both the vaccination policy and the domestically implemented strain.

## Figures and Tables

**Table 1:** Baseline Characteristics of the Study Population (N = 3035).

Variables	N (%) or Median [IQR]
**Demographics and Clinical Characteristics**	
Male sex	1587 (52.3)
Age at MMR + V vaccination (months)	12 [12, 12]
Presence of complex chronic conditions	412 (13.6)
Total healthcare encounters during observation	9 [6, 14]
**Vaccination Sequences and Timing**	
Sequential order of study vaccines	
MMR + V followed by HAV	2790 (91.9)
HAV followed by MMR + V	245 (8.1)
Interval between MMR + V and HAV (days)	17 [7, 63]
**Immunization Intensity (Vaccine Burden)**	
Total vaccines administered at MMR + V visit	
2 vaccines (MMR and varicella only)	1848 (60.9)
≥3 vaccines (≥1 additional vaccine *)	1187 (39.1)
Total vaccines during 42-day post-MMR + V period	6 [5, 7]
During MMR + V-dominant period	3 [2, 5]
During Overlap period	3 [2, 4]
**Vaccine and Environmental Factors**	
Varicella vaccine strain (MAV/06)	2175 (71.7)
Pandemic phase (2015–2019, prepandemic)	1851 (61.0)

Abbreviations: HAV, hepatitis A vaccine; IQR, interquartile range; MMR + V, concurrent measles–mumps–rubella and varicella vaccination. * Most frequent additional vaccine: Japanese encephalitis vaccine (37.0%). Bold indicates variable category subheadings.

**Table 2:** Incidence Rates and Adjusted Relative Risks of Adverse Events Following MMR + V and HAV Vaccinations.

Outcome and Exposure State	Events (n)	IR ^a^	aRR ^b^ (95% CI)	*p* Value
**Fever**				
Baseline	725	1.05	Reference	—
MMR + V-dominant	165	2.28	2.43 (1.57–3.74)	<0.001 *
Overlap	88	1.51	1.29 (0.95–1.75)	0.106
HAV-dominant	68	0.94	1.03 (0.77–1.39)	0.838
MMR + V-dominant vs. overlap	—	—	1.88 (1.11–3.20)	0.019 *
MMR + V-dominant vs. HAV-dominant	—	—	2.35 (1.39–3.98)	0.001 *
**Total sick visits ^c^**				
Baseline	3427	4.95	Reference	—
MMR + V-dominant	575	7.95	1.16 (0.93–1.45)	0.192
Overlap	359	6.17	1.04 (0.89–1.21)	0.640
HAV-dominant	323	4.47	0.81 (0.70–0.94)	0.005 *
MMR + V-dominant vs. overlap	—	—	1.12 (0.85–1.47)	0.412
MMR + V-dominant vs. HAV-dominant	—	—	1.43 (1.09–1.87)	0.009 *
**Acute care visits ^c^**				
Baseline	1381	2.00	Reference	—
MMR + V-dominant	205	2.83	1.09 (0.76–1.55)	0.649
Overlap	144	2.47	1.00 (0.79–1.27)	0.986
HAV-dominant	118	1.63	0.77 (0.61–0.98)	0.032 *
MMR + V-dominant vs. overlap	—	—	1.08 (0.71–1.66)	0.714
MMR + V-dominant vs. HAV-dominant	—	—	1.40 (0.92–2.15)	0.116
**Febrile convulsions**				
Baseline	46	0.07	Reference	—
MMR + V-dominant	8	0.11	1.17 (0.15–8.84)	0.882
Overlap	4	0.07	1.13 (0.27–4.73)	0.866
HAV-dominant	5	0.07	0.96 (0.26–3.52)	0.950
MMR + V-dominant vs. overlap	—	—	1.03 (0.09–12.30)	0.981
MMR + V-dominant vs. HAV-dominant	—	—	1.22 (0.11–13.49)	0.874
**Allergic reactions**				
Baseline	166	0.24	Reference	—
MMR + V-dominant	25	0.35	1.60 (0.59–4.33)	0.359
Overlap	15	0.26	0.72 (0.35–1.50)	0.385
HAV-dominant	19	0.26	1.53 (0.79–2.97)	0.207
MMR + V-dominant vs. overlap	—	—	2.20 (0.64–7.56)	0.210
MMR + V-dominant vs. HAV-dominant	—	—	1.04 (0.31–3.45)	0.947
**Rash**				
Baseline	58	0.08	Reference	—
MMR + V-dominant	17	0.24	2.06 (0.45–9.37)	0.350
Overlap	12	0.21	2.01 (0.76–5.29)	0.157
HAV-dominant	6	0.08	0.91 (0.33–2.50)	0.850
MMR + V-dominant vs. overlap	—	—	1.02 (0.17–6.18)	0.979
MMR + V-dominant vs. HAV-dominant	—	—	2.27 (0.37–14.07)	0.378

Abbreviations: aRR, adjusted relative risk; CI, confidence interval; HAV, hepatitis A vaccine; IR, incidence rate; MMR + V, concurrent measles–mumps–rubella and varicella vaccination. * Statistically significant (*p* < 0.05). ^a^ Calculated based on total person-days of the entire cohort (Baseline: 692,002; MMR + V-dominant: 72,327; HAV-dominant: 72,327; Overlap: 58,196) and applied consistently across all outcomes. ^b^ Adjusted for age, season, the number of concomitant vaccines administered within each exposure period, and the pandemic period. Estimated using only informative cases (subjects with ≥1 event). The number of subjects contributing to each model varied by outcome. ^c^ Total sick visits represent all acute clinical encounters. Acute care visits, a subset of total sick visits, were defined as encounters requiring emergency department visits or hospitalization. Bold indicates outcome category subheadings.

## Data Availability

The data supporting the findings of this study are available from the corresponding author upon reasonable request.

## Supplementary Materials

The following supporting information can be downloaded at: https://www.mdpi.com/article/10.3390/vaccines14070553/s1; Supplementary Methods; Supplementary Table S1: Definitions of Adverse Events of Interest and Clinical Outcomes Using ICD-10 Codes; Supplementary Table S2: Temporal trends in adjusted relative risks for major clinical outcomes following MMR+V vaccination; Supplementary Table S3: Adjusted Relative Risks of Adverse Events Following MMR+V and HAV Vaccinations: Subgroup Analysis of the MAV/06 Varicella Strain.
