# Supplementary material for: Real-World Safety of Concurrent Measles–Mumps–Rubella and Varicella Vaccination in Korean Infants: A Multicenter Self-Controlled Case Series Study
Source: Vaccines (Basel). 2026 Jun 24;14(7):553. doi: 10.3390/vaccines14070553 (PMC13418243; doi:10.3390/vaccines14070553)
Supplement: Supplementary file 1 [file vaccines-14-00553-s001.zip › vaccines-4355954-supplementary.pdf]

Supplementary Material

Supplementary Methods

Supplementary Table S1. Definitions of Adverse Events of Interest and Clinical Outcomes Using ICD-10 Codes

Supplementary Table S2. Temporal trends in adjusted relative risks for major clinical outcomes following MMR+V vaccination.

Supplementary Table S3. Adjusted Relative Risks of Adverse Events Following MMR+V and HAV Vaccinations: Subgroup Analysis of the MAV/06 Varicella Strain

Supplementary Methods

Clinical Outcome Definition and Data Cleaning

To identify predefined adverse events (AEs)—including allergic reactions, rash, fever, febrile convulsion, and sick visits—International Classification of Diseases, 10th Revision (ICD-10) codes were extracted from electronic health record (EHR). The complete list of diagnostic codes is provided in Supplementary Table S1.

A multi-step cleaning algorithm was implemented to ensure the independence of clinical events and to minimize overestimation from follow-up care:

- Episode Definition and Washout Period: For each subject, a 42-day washout period was applied to each clinical department. Any medical encounter sharing the same ICD-10 code within 42 days of a previous visit was flagged as a redundant episode. For the SCCS analysis, only the first encounter date of each independent episode was counted as

an outcome.

- Hierarchical Classification of Sick Visits:
    - Acute care visits: All emergency department (ED) visits or hospitalizations coded with predefined acute illness codes were classified as acute care visits.
    - Outpatient visits: An outpatient "sick visit" was defined as the first encounter involving a target ICD-10 code (Supplementary Table S1) as the primary diagnosis, provided it had not been recorded in the preceding 42 days.
- Exclusion Criteria: To isolate vaccine-related AEs, encounters primarily coded for injuries, poisoning, or other external causes (ICD-10 codes S00–T98) and congenital anomalies (Q00–Q99) were excluded. However, specific codes representing adverse effects or complications related to therapeutic substances and immunization—including poisoning by drugs (T36–T50, T96), adverse effects not elsewhere classified (T78), and complications following infusion or therapeutic injection (T80, T88)—were retained. Routine clinical encounters solely for vaccination or well-child checks were not counted as sick visits.

Supplementary Table S1. Definitions of Adverse Events of Interest and Clinical Outcomes Using ICD-10 Codes

Adverse Event of Interest	ICD-10 Codes
Fever	R508, R509, R5090, R5099
Febrile Convulsion	R560, or R568/R568A recorded in conjunction with a concurrent fever code.
Allergic Reactions	L500, L501, L509, L5088, T783, T784, T784A
Rash	R21
Sick visits	All clinical encounters associated with the specific adverse events defined above (fever, febrile convulsion, allergic reactions, and rash), plus a broader range of acute diagnostic codes including respiratory, gastrointestinal, and other infectious diseases:<br><br>A020, A047, A049, A052, A080, A081, A0838, A084, A084A, A090, A090E, A090G, A090J, A099, A38, A411, A411A, A4150, A4158, A419, A491, A493, A870, B001A, B001B, B002, B004, B0052, B0088, B009, B019, B07, B081, B082, B084, B084A, B085, B085A, B09B, B302, B340, B341, B348, B3488, B349, B970, B974, B99, E860, E162, G002, G009, G039, G4090, H050B, H103, H6038, H6088, H609, H6500, H6501, H66000 H6640, H6690, H6690A, H6691,

H6691A H920, I880, I889, J00, J00A, J00D, J0190, J029, J029B, J0380, J0390, J0390A J040, J041, J042, J050, J060, J068, J069, J100, J101, J101A, J108, J108B, J111, J118, J120, J121, J122, J123, J1280, J1288, J129, J157, J180, J181, J188, J189, J200, J204, J205, J206, J2080, J2088, J209, J210, J211, J2180, J2181, J219, J302, J340, J340A, J348, J40, K1120, K121A, K121C, K121D, K296, K599, K561, K561A, K567, K752, K759, K831, K759A, K921, K922B, L0108, L0109, L0240, L0241H L0290, L0300, L0310C L0332, L039, L040, L048, L049, L089, L282, L299, L303, L309, L444, L503, L519, L539, L539A, M0096A M1285A M2552, M2555, M2556, M2557, M303, M5450, M7967, N10, N10B, N10C, N390, R000, R05, R060, R093D, R100, R102, R1039, R1049, R112, R113, R14, R14A, R220, R221, R223, R224, R229, R230, R300, R454, R51, R520, R53, R572, R630, R681B, T782, T782B, T801, T881, T881A, U071, U072

Supplementary Table S2. Temporal trends in adjusted relative risks for major clinical outcomes following MMR+V vaccination.

Outcome and Interval following MMR+V	aRR [a] (95% CI)	P-value
**Fever**		
Baseline	Reference	-
0 – 6 days	1.14 (0.66 - 1.98)	0.639
7 – 13 days	4.27 (2.76 - 6.60)	**<0.001***
14 – 20 days	0.78 (0.43 - 1.43)	0.430
21 – 27 days	0.58 (0.30 - 1.13)	0.109
28 – 42 days	0.78 (0.50 - 1.21)	0.270
**Total sick visits** [b]		
Baseline	Reference	-
0 – 6 days	0.59 (0.43 - 0.82)	**<0.001***
7 – 13 days	2.15 (1.70 - 2.71)	**<0.001***
14 – 20 days	0.70 (0.52 - 0.93)	**0.015***
21 – 27 days	0.69 (0.52 - 0.91)	**0.010***
28 – 42 days	1.00 (0.82 - 1.21)	0.976
**Acute care visits** [b]		

Baseline	Reference	-
0 – 6 days	0.74 (0.46 - 1.19)	0.213
7 – 13 days	2.13 (1.46 - 3.10)	**<0.001***
14 – 20 days	0.63 (0.39 - 1.03)	0.065
21 – 27 days	0.53 (0.32 - 0.89)	**0.015***
28 – 42 days	0.96 (0.70 - 1.33)	0.818
**Allergic reactions**		
Baseline	Reference	-
0 – 6 days	1.20 (0.46 - 3.16)	0.708
7 – 13 days	1.37 (0.53 - 3.55)	0.515
14 – 20 days	0.90 (0.31 - 2.61)	0.847
21 – 27 days	1.13 (0.42 - 3.03)	0.811
28 – 42 days	0.54 (0.21 - 1.36)	0.192
**Rash**		
Baseline	Reference	-
0 – 6 days	1.86 (0.40 - 8.71)	0.432
7 – 13 days	5.29 (1.56 - 17.94)	**0.008***
14 – 20 days	5.23 (1.60 - 17.08)	**0.006***
21 – 27 days	2.85 (0.81 - 10.08)	0.104

28 – 42 days	0.79 (0.20 - 3.09)	0.74
**Febrile convulsion**		
Baseline	Reference	-
0 – 6 days	0.59 (0.05 - 6.40)	0.665
7 – 13 days	5.37 (1.20 - 24.01)	**0.028***
14 – 20 days	1.65 (0.25 - 10.78)	0.599
21 – 27 days	0.77 (0.08 - 7.38)	0.818
28 – 42 days	0.33 (0.04 - 2.96)	0.324

Abbreviations: aRR, adjusted relative risk; CI, confidence interval; HAV, hepatitis A vaccine; MMR+V, measles-mumps-rubella + varicella vaccines.

Note: Asterisks indicate statistical significance at a *P*-value less than 0.05.

[a] Adjusted for sex, age, presence of complex chronic condition, season and pandemic period. Estimated using only informative cases (subjects with >=1 event). The number of subjects contributing to each model varies by outcome.

[b] Total sick visits represent all acute clinical encounters. Acute care visits, a subset of total sick visits, were defined as encounters requiring emergency department encounters or hospitalizations.

Supplementary Table S3. Adjusted Relative Risks of Adverse Events Following MMR+V and HAV Vaccinations: Subgroup Analysis of the MAV/06 Varicella Strain

Outcome and Exposure State	Events (n)	IR[a]	aRR[b] (95% CI)	P-value
**Fever**				
Baseline	597	1.20	Reference	-
MMR+V-dominant	136	2.89	2.07 (1.28 - 3.34)	**0.003**[*]
Overlap	75	1.61	1.11 (0.79 - 1.56)	0.536
HAV-dominant	49	1.04	0.88 (0.62 - 1.24)	0.472
MMR+V-dominant vs overlap	—	—	1.86 (1.03 - 3.34)	**0.038**[*]
MMR+V-dominant vs HAV-dominant	—	—	2.35 (1.30 - 4.24)	**0.005**[*]
**Total sick visits** [c]				
Baseline	2,855	5.76	Reference	-
MMR+V-dominant	446	9.49	0.97 (0.76 - 1.25)	0.841
Overlap	308	6.62	0.90 (0.76 - 1.06)	0.210
HAV-dominant	264	5.62	0.80 (0.68 - 0.94)	**0.007**[*]
MMR+V-dominant vs overlap	—	—	1.08 (0.80 - 1.47)	0.598
MMR+V-dominant vs HAV-dominant	—	—	1.22 (0.90 - 1.64)	0.194
**Acute care visits** [c]				

Baseline	1,159	2.34	Reference	-
MMR+V-dominant	162	3.45	0.85 (0.57 - 1.26)	0.414
Overlap	117	2.51	0.80 (0.61 - 1.05)	0.103
HAV-dominant	96	2.04	0.72 (0.55 - 0.93)	**0.014***
MMR+V-dominant vs overlap	—	—	1.06 (0.66 - 1.71)	0.813
MMR+V-dominant vs HAV-dominant	—	—	1.18 (0.73 - 1.90)	0.496
**Febrile Convulsion**				
Baseline	28	0.06	Reference	-
MMR+V-dominant	4	0.09	0.46 (0.03 - 7.58)	0.587
Overlap	2	0.04	0.63 (0.09 - 4.32)	0.635
HAV-dominant	4	0.09	0.80 (0.15 - 4.26)	0.795
MMR+V-dominant vs overlap	—	—	0.73 (0.02 - 22.07)	0.859
MMR+V-dominant vs HAV-dominant	—	—	0.57 (0.02 - 14.97)	0.738
**Allergic reaction**				
Baseline	88	0.18	Reference	-
MMR+V-dominant	17	0.36	2.63 (0.74 - 9.37)	0.136
Overlap	9	0.19	0.96 (0.36 - 2.55)	0.935
HAV-dominant	12	0.26	1.92 (0.86 - 4.31)	0.112
MMR+V-dominant vs overlap	—	—	2.74 (0.55 - 13.61)	0.218

MMR+V-dominant vs HAV-dominant	—	—	1.37 (0.30 - 6.16)	0.684
**Rash**				
Baseline	48	0.10	Reference	-
MMR+V-dominant	8	0.17	0.98 (0.11 - 8.66)	0.985
Overlap	10	0.21	1.56 (0.46 - 5.31)	0.481
HAV-dominant	5	0.11	1.07 (0.32 - 3.61)	0.912
MMR+V-dominant vs overlap	—	—	0.63 (0.05 - 7.68)	0.717
MMR+V-dominant vs HAV-dominant	—	—	0.91 (0.08 - 11.08)	0.944

Abbreviations: IR, incidence rate; aRR, adjusted relative risk; CI, confidence interval; HAV, hepatitis A vaccine; MMR+V, measles-mumps-rubella + varicella vaccines.

Note: Asterisks indicate statistical significance at a *P*-value less than 0.05.

[a] Calculated based on the fixed total person-days accumulated within each respective exposure state for the MAV/06 subcohort.

[b] Adjusted for age, season and pandemic period. Estimated using only informative cases (subjects with ≥1 event). The number of subjects contributing to each model varies by outcome.

[c] Total sick visits represent all acute clinical encounters. Acute care visits, a subset of total sick visits, were defined as encounters requiring emergency department encounters or hospitalizations.
